# Worldwide anaesthesia use during endovascular treatment for medium vessel occlusion stroke

**DOI:** 10.1177/15910199211041487

**Published:** 2021-10-19

**Authors:** Manon Kappelhof, Johanna M Ospel, Petra Cimflova, Nima Kashani, Nishita Singh, Rosalie McDonough, Arshia Sehgal, Mohammed A Almekhlafi, Jens Fiehler, Michael Chen, Nobuyuki Sakai, Charles BLM Majoie, Mayank Goyal

**Affiliations:** 1 Department of Diagnostic Imaging, Foothills Medical Center, 70401 University of Calgary, Canada; 2 Department of Radiology and Nuclear Medicine, 26066 Amsterdam University Medical Centers, University of Amsterdam, the Netherlands; 3 Department of Radiology, 30262 University Hospital of Basel, Switzerland; 4 Department of Clinical Neurosciences, 2129 University of Calgary, Canada; 5 Department of Neuroradiology, University Medical Center Hamburg-Eppendorf, Germany; 6 Department of Neurological Sciences, 2468 Rush University Medical Center, USA; 7 Department of Neurosurgery, Kobe City Medical Center General Hospital, Japan

**Keywords:** Ischemic stroke, anaesthesia, thrombectomy

## Abstract

**Introduction:**

The optimal anaesthesia approach for endovascular treatment (EVT) in acute ischaemic stroke is currently unknown. In stroke due to medium vessel occlusions (MeVO), the occluded vessels are particularly small and more difficult to access, especially in restless or uncooperative patients. In these patients, general anaesthesia (GA) may be preferred by physicians to prevent complications due to patient movement. We investigated physicians’ approaches to anaesthesia during EVT for MeVO stroke.

**Methods:**

In a worldwide, case-based, online survey, physicians’ preferred anaesthesia approach during EVT for MeVO stroke was categorized as “initial GA”, “initial GA if necessary” (depending on patient cooperation), “no initial GA, but conversion if necessary” (start with local anaesthesia or conscious sedation), and “no GA”. Preferred anaesthesia approaches were reported overall and stratified by physician and patient characteristics.

**Results:**

A total of 366 survey participants provided 1464 responses to 4 primary MeVO EVT case-scenarios. One-third of responses (489/1464 [33%]) favoured no initial GA, but conversion if necessary. Both initial GA and initial GA if necessary were preferred in 368/1464 (25%) of responses respectively. No GA was favoured in 244/1464 (17%). Occlusion location, respondent specialization (interventional neuroradiology), higher age, and female respondent sex were significantly associated with GA preference. GA was more often used in Europe than in other parts of the world (*p* < 0.001).

**Conclusions:**

Anaesthesia approaches in MeVO EVT vary across world regions and patient and physician factors. Most physicians in this survey preferred to start with local anaesthesia or conscious sedation and convert to GA if necessary.

## Introduction

The optimal approach to anaesthesia during endovascular treatment (EVT) for acute ischaemic stroke remains unclear despite several randomized single-centre trials.^1^ Current guidelines provide no clear recommendations due to this lack of evidence,^2^ and physicians’ approaches to peri-procedural anaesthesia during EVT for large vessel occlusions (LVO) vary across the world.^3^

When EVT is performed in patients with medium vessel occlusions (MeVO; e.g. occlusions of the [distal] M2/M3, A2/A3, or P2/P3 vessel segments),^4^ anaesthesia may be of additional importance: the occluded vessels are smaller and potentially more difficult to access compared to those in LVO stroke, especially in restless or uncooperative patients. General anaesthesia (GA) may then increase procedural safety in MeVO stroke by reducing patient motion.^5^ Of note, the benefit of EVT in MeVO stroke has not been proven in randomized trials, though observational studies suggest substantial benefit.^4,6^

Currently used approaches to anaesthesia during EVT for MeVO stroke are thus not dictated by guidelines, but they are highly relevant for future randomized studies. Therefore, we aimed to assess stroke physicians’ anaesthesia preferences for EVT in MeVO strokes as part of a worldwide case-based survey: MeVO – Finding Rationales and Objectifying New Targets for IntervEntional Revascularization in Stroke (MeVO-FRONTIERS).

## Materials and methods

### Survey design

We conducted a clinical case-vignette-based, international, online, cross-sectional survey to assess stroke physicians’ current practice and preferences with regard to EVT for MeVO stroke.^7^ The survey consisted of seven cases, each comprising a brief clinical case-vignette and exemplary computed tomography (CT) or angiography imaging findings. Four of these cases were primary MeVOs, three were secondary MeVOs, i.e. MeVOs that resulted from thrombus migration of an initial LVO.^8^ At the end of each case, respondents were asked for their preferred anaesthesia approach. In the current study, we evaluate the responses to these questions. All case-vignettes and anaesthesia questions are shown in Appendix A.

The survey was distributed through Qualtrics (www.qualtrics.com) from 12 November 2020 through 31 December 2020 and took approximately 30 minutes to complete. All collected data were anonymous. Case-scenarios described combined data from real and fictional patients, and could not be traced back to individual patients. The Conjoint Health Research Ethics Board of the University of Calgary approved this study (REB20-2086). Data are available from the corresponding author upon reasonable request

### Survey participants

The survey was distributed to approximately 1400 physicians involved in acute stroke care through professional neuro-interventional and stroke associations and the personal and professional networks of the investigators. Survey respondents’ baseline demographics (specialization, age range, sex, region of practice, range of personal and institutional EVT cases per year, hospital setting, hospital 24/7 EVT coverage, range of years of experience and career stage) were collected prior to answering the case-scenarios. There were no restrictions on participation in the survey for any of these variables.

### Anaesthesia preference

For primary MeVO case-scenarios (*n* = 4), answer options were as follows: (a) *I do all EVTs under GA*; (b) *I do all MeVO EVTs under GA*; (c) *If this patient was uncooperative I would use GA (lower threshold than for large vessel occlusions)*; (d) *If this patient was uncooperative I would use GA (same threshold as for large vessel occlusions)*; (e) *I do all EVTs under local anaesthesia/conscious sedation (LA/CS)*; (f) *I would start under LA/CS but convert to GA if the patient is uncooperative (lower threshold than for large vessel occlusions)*; (g) *I would start under LA/CS but convert to GA if the patient is uncooperative (same threshold as for large vessel occlusion)*.

For the current analysis, these were categorized as (1) “initial GA” (options a and b), (2) “initial GA if necessary” (depending on patient cooperation; c and d), (3) “no initial GA, but conversion if necessary” (start with local anaesthesia/conscious sedation; f and g) and (4) “no GA” (option e).

For secondary MeVO scenarios (*n* = 3), the primary occlusion site was shown first (on CT or angiography imaging) followed by the secondary occlusion site (on angiography imaging). Physicians were then asked if they would convert to GA. Possible answers were (a) *Yes* (convert to GA), (b) *If the patient was even slightly uncooperative (lower threshold than for large vessel occlusion), I would convert to general anaesthesia*, (c) *If the patient was uncooperative (same threshold as for large vessel occlusion), I would convert to general anaesthesia*, (d) *No* (I do not convert to GA).

These were summarized as (1) “conversion to GA” (option a), (2) “conversion to GA if necessary” (depending on patient cooperation; b and c), and (3) “no conversion to GA” (option d). The exact medication used for inducing and maintaining GA or conscious sedation was not specified.

### Statistical analyses

Baseline demographics were described as appropriate to the type of data. In the descriptive analyses, additional stratified results are presented based on world region of practice and occlusion location. Univariable and multivariable ordinal logistic regression analyses were performed to assess the effect of occlusion location and respondent demographics on anaesthesia preference for the primary MeVO cases. Variables were included in the multivariable model if they were associated with anaesthesia choice in the univariable analysis with *p* < 0.1. Anaesthesia preference was treated as an ordinal variable increasing from ‘no GA’ to ‘initial GA’. For the secondary MeVO cases, results were presented descriptively, and no regression analyses were performed to limit the number of statistical tests and decrease the risk of type 1 error. The significance level was set at *p* < 0.05. Analyses were performed in Stata 14.1 (StataCorp, TX, USA).

## Results

Three-hundred-sixty-six physicians provided 1464 answers to four primary MeVO EVT case-scenarios, and 1098 answers to three secondary MeVO case-scenarios (Supplemental Table S1). Of all respondents, 84% were male, and 91% worked in hospitals with 24/7 EVT coverage.

### Primary MeVO

**Figure 1.**
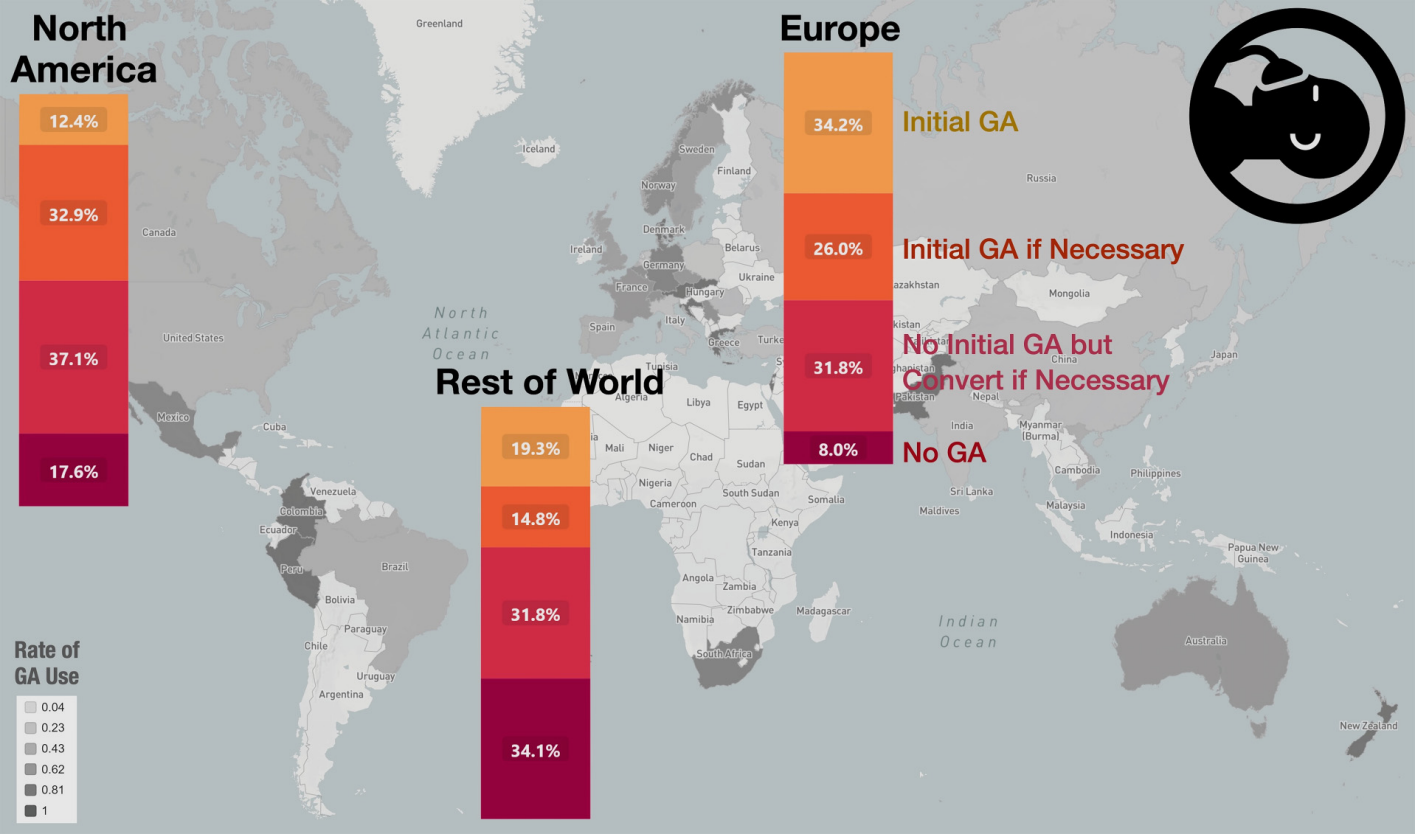
Anaesthesia preference by region of practice. Regions are divided into Europe (*n* = 179 respondents, 49%), North America (*n* = 95, 26%), and the rest of the world (Asia, Pacific and Africa: *n* = 79, 22%; South America: *n* = 13, 4%). GA: general anaesthesia.

**Figure 2.**
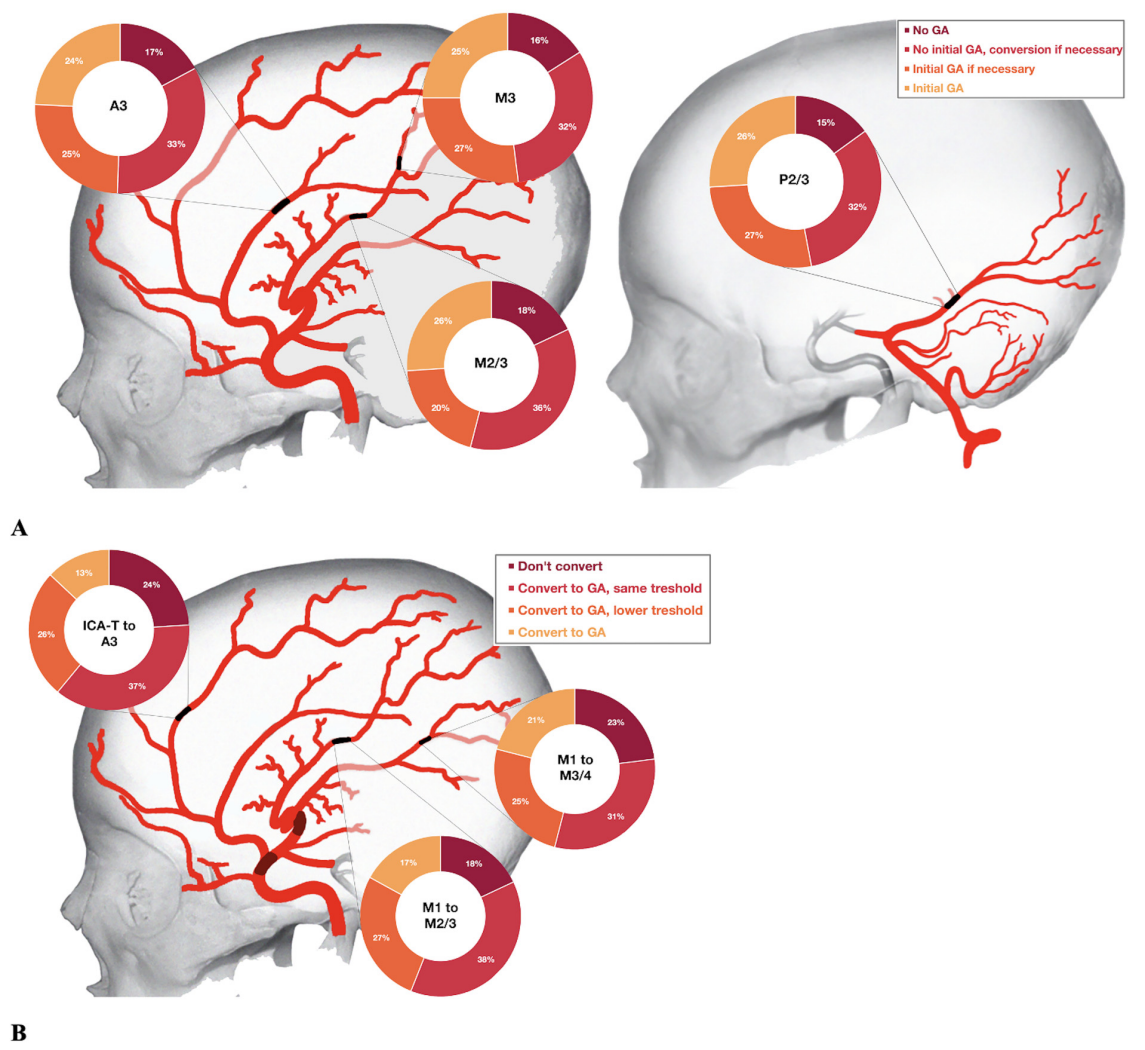
Anaesthesia preference by occlusion location for primary (A) and secondary (B) MeVOs. A 3: third segment of anterior cerebral artery; GA: general anaesthesia; ICA-T: internal carotid artery terminus; M1/M2/M3: first/second/third segments of middle cerebral artery; P2/3: second/third segment of posterior cerebral artery.

One-third (489/1464 responses) favoured no initial GA, but conversion if necessary. Seventeen percent (244/1464) opted for no GA, 25% (363/1464) chose initial GA if necessary based on patient cooperation, and 25% (368/1464) opted for initial GA. Preference for GA was highest among interventional neuroradiologists (245/680 responses [36%]), in physicians 41–50 years old (30%) and in those with >10 years of neuro-interventional experience (33%) (Table 1; Supplemental Table S2). GA was used more often in Europe than in other parts of the world (Figure 1, *p* *<* 0.001). Anaesthesia preference also varied by occlusion location (Figure 2(A)). In the multivariable regression analyses, physician age (acOR per decade increase: 1.19, 95%CI: 1.02–1.40), female physician sex (acOR 1.63, 95% CI: 1.16–2.29), and respondent specialization (acOR 0.78, 95% CI: 0.69–0.89) were significantly associated with preference for GA (Supplemental Table S3).

**Table 1.** Respondent and case characteristics per preferred anaesthesia approach for primary MeVO cases.

		No GA (*n* = 244)	No initial GA, but conversion if necessary^a^ (*n* = 489)	Initial GA if necessary^a^ (*n* = 363)	Initial GA (*n* = 368)	Unadjusted cOR (95% CI)^b^ *Univariable models*
Case occlusion location – *n(*%*)*						**1.03 (1.01–1.05)**
	M2/3	65 (18)	133 (36)	74 (20)	94 (26)	
	M3	60 (16)	116 (32)	98 (27)	92 (25)	
	A3	64 (17)	122 (33)	92 (25)	88 (24)	
	P2/3	55 (15)	118 (32)	99 (27)	94 (26)	
Respondent specialization – *n(*%*)*						**0.78 (0.69–0.89)**
	Interventional Neuroradiologist	78 (11)	184 (27)	173 (25)	245 (36)	
	Interventional radiologist	20 (28)	38 (53)	14 (19)	0 (0)	
	Neurosurgeon	44 (28)	61 (39)	15 (10)	36 (23)	
	Neurologist	102 (19)	195 (37)	153 (29)	82 (15)	
Respondent sex – *n(*%*)*						**1.45 (1.04–2.02)**
	Male	231 (19)	415 (34)	26 (22)	317 (26)	
	Female	13 (6)	74 (33)	86 (38)	51 (23)	
Respondent age – *n(*%*)*						**1.22 (1.04–1.43)**
	<40years	59 (15)	164 (41)	116 (29)	65 (16)	
	41–50years	121 (20)	187 (31)	112 (19)	184 (30)	
	51–60years	5 (18)	106 (33)	86 (27)	71 (22)	
	>60years	7(5)	32 (24)	49(36)	44 (33)	
Neuro-interventional experience – *n(*%*)*						1.07 (0.93–1.24)
	<10 years	83 (16)	185 (35)	159 (30)	101 (19)	
	>10 years	131 (18)	218 (30)	142 (20)	237 (33)	
Region of practice – *n(*%*)*						1.08 (0.95–1.23)
	Europe	57 (8)	288 (32)	186 (26)	245 (34)	
	North America	67 (18)	141 (37)	125 (33)	47 (12)	
	Rest of the world	120 (33)	120 (33)	52 (14)	76 (21)	

Additional variables shown in Supplemental Table S2. A3: third segment of anterior cerebral artery; CI: confidence interval; cOR: common odds ratio; EVT: endovascular treatment; GA: general anaesthesia; M2/3: second/third segment of middle cerebral artery; P2/3: second/third segment of posterior cerebral artery.

^a^ If necessary, based on patient cooperativeness.

^b^ Univariable ordinal logistic regression models with GA preference as ordinal outcome (from 0 – no GA to 3 – initial GA), clustered for respondent and scenario number. If *p* < 0.01 (age, sex, specialization), variables were included in the ultivariable model: reported in the Results section text.

### Secondary MeVO

Most responses indicated that they would convert to GA if necessary (675/1098, 61%). Of those, 389 would convert using the same threshold as for LVO stroke. Table 2 shows respondent and case-scenario characteristics over all responses. Conversion to GA if necessary was the most commonly chosen approach, irrespective of physicians and case-scenario characteristics. The proportion of physicians preferring direct conversion to GA was higher in Europe (123/537 responses, 23%) than in North America (21/285 responses, 7%), and among physicians aged >60 years (27/102, 26%) compared to their younger colleagues (14%–19%). In addition, more physicians preferred GA in M3/4 occlusions than M2/3 or A3 occlusions (Figure 2(B)).

**Table 2.** Respondent and case characteristics per preferred anaesthesia approach for secondary MeVO cases.

		Convert to GA (*n* = 186)	Convert to GA if necessary^a^ (*n* = 675)	No conversion to GA (*n* = 237)
Case occlusion location – *n(*%*)*				
	M2/3	62 (17)	238 (65)	66 (18)
	M3/4	77 (21)	205 (56)	84 (23)
	A3	47 (13)	232 (63)	87 (24)
Respondent specialization – *n(*%*)*				
	Interventional Neuroradiologist	112 (22)	305 (60)	14 (26)
	Interventional Radiologist	2 (4)	38 (70)	14 (26)
	Neurosurgeon	23 (20)	58 (50)	36 (31)
	Neurologist	29 (10)	207 (71)	55 (19)
Respondent sex – *n(*%*)*				
	Male	159 (17)	552 (60)	213 (23)
	Female	27 (16)	119 (71)	22 (13)
Respondent age – *n(*%*)*				
	<40years	41 (14)	202 (67)	60 (21)
	41–50years	84 (19)	255 (56)	114 (25)
	51–60years	34 (14)	154 (64)	52 (22)
	>60years	27 (26)	64 (63)	11 (11)
Neuro-interventional experience – *n(*%*)*				
	<10 years	57 (14)	254 (64)	85 (21)
	>10 years	129 (18)	421 (60)	152 (22)
Region of practice – *n(*%*)*				
	Europe	123 (23)	347 (64)	67 (12)
	North America	21 (7)	197 (69)	67 (24)
	Rest of the world	42 (15)	131 (48)	103 (37)

Additional variables shown in Supplemental Table S2. A3: third segment of anterior cerebral artery; EVT: endovascular treatment; GA: general anaesthesia; M2/3: second/third segment of middle cerebral artery; P2/3: second/third segment of posterior cerebral artery.

^a^ If necessary, based on patient cooperativeness (lower threshold than for large vessel occlusion: *n* = 286, same threshold as for large vessel occlusion: *n* = 389).

## Discussion

In this study, one-third of stroke physicians preferred to start with local anaesthesia/conscious sedation when performing EVT for MeVO stroke and would convert to GA if necessary. Initial GA and initial GA if necessary based on patient cooperation, were the second-most frequent choices (each 25%). However, preferences varied by world region and by physician age, sex, and specialization.

In LVO stroke, observational studies found worse clinical outcomes with EVT under GA, while single-centre randomized trials have reported non-inferior outcomes.^1^ Keeping patient motion at a minimum to reduce the risk of complications seems more important in EVT for MeVO stroke compared to LVO stroke.^5^ Thus, one would expect a higher use of GA during MeVO EVT. Interestingly, physicians in the present study mostly favoured local anaesthesia/conscious sedation over GA for MeVO EVT, and as such, which resulted in lower use of GA compared to previous studies.^9,10^ Comparability to our results is however limited, as these studies were not centred around MeVOs and conversion from initial conscious sedation/local anaesthesia to GA was not captured in detail.

As for secondary MeVOs, the proportion of physicians who would convert to GA was lower (13–21% depending on the occlusion site, Figure 2(B)) compared to those who would start the procedure under GA in primary MeVOs (24–26%, Figure 2(A)), while more physicians opted against GA in secondary MeVOs (18–24% vs. 15–18% in primary MeVOs, Figure 2). This could be related to the fact that conversion to GA in the middle of the procedure is more difficult to organize than the initiation of GA prior to the procedure, and may substantially prolong procedure time.

Anaesthesia preferences for MeVO EVT in our study differed significantly between world regions, with European physicians leaning more towards the use of GA, which confirms the results of a previous survey-based study.^9^ Access to timely anaesthesia may vary between healthcare systems: while in some countries, an anaesthesiologist is always available in the neuro-angiography suite, this may not be the case in others, which would potentially result in substantial time delays. The differences we observed related to physician characteristics should be considered purely exploratory and hypothesis-generating. Possibly, the higher use of GA in European countries is associated with a higher rate of interventional neuroradiologists performing EVT. These observations may serve as a starting point for future investigations.

### Limitations

First, by distributing the survey through professional and personal networks, selection bias may have occurred. Specifically, the number of female interventionists in our study was low. Second, with 366 complete responses out of approximately 1400 physicians approached, our response rate was relatively low. The number of responses and length of the survey constitute a trade-off in survey design: with a median duration of 30 min, the length of our survey may at least partially explain this low response rate. Third, we did not ask or specify the availability of anaesthesiologists, though this might influence physicians’ anaesthesia preferences. Finally, the images we provided with the case-scenarios showed a specific vascular anatomy, which may not be generalizable to the entire MeVO stroke population.

## Conclusions

Anaesthesia approaches in MeVO EVT vary widely across world regions and are based on patient and physician factors. One-third of physicians in this survey preferred to start with local anaesthesia/conscious sedation and convert to GA if necessary.

## Abbreviations

MeVO: medium vessel occlusion
EVT: endovascular treatment
LVO: arge vessel occlusion
CT: computed tomography
GA: general anaesthesia
CI: confidence interval
(a)(c)OR: (adjusted)(common) odds ratio
